# Repeated exposure to dengue virus elicits robust cross neutralizing antibodies against Zika virus in residents of Northeastern Thailand

**DOI:** 10.1038/s41598-021-88933-x

**Published:** 2021-05-05

**Authors:** Sararat Hattakam, Annie Elong Ngono, Melanie McCauley, Sujan Shresta, Montarop Yamabhai

**Affiliations:** 1 Molecular Biotechnology Laboratory, School of Biotechnology, Institute of Agricultural Technology, Suranaree University of Technology, Nakhon Ratchasima, 30000 Thailand; 2 Center for Infectious Disease and Vaccine Research, La Jolla Institute for Immunology, La Jolla, CA 92037 USA; 3 Department of Medicine, University of California, San Diego, La Jolla, CA 92161 USA

**Keywords:** Viral infection, Viral infection

## Abstract

Zika virus (ZIKV) and dengue virus (DENV) are antigenically related mosquito-borne flaviviruses. ZIKV is becoming increasingly prevalent in DENV-endemic regions, raising the possibility that pre-existing immunity to one virus could modulate the response to a heterologous virus, although whether this would be beneficial or detrimental is unclear. Here, we analyzed sera from residents of a DENV-endemic region of Thailand to determine the prevalence of DENV-elicited antibodies capable of cross-neutralizing ZIKV. Sixty-one participants who were asymptomatic and unselected for viral serostatus were enrolled. Among them, 52 and 51 were seropositive for IgG antibody against DENV or ZIKV E proteins (ELISA assay), respectively. Notably, 44.23% (23/52) of DENV seropositive participants had serological evidence of multiple exposures to DENV, and these subjects had strikingly higher titers and broader reactivities of neutralizing antibodies (NAbs) against ZIKV and DENV heterotypes compared with participants with serological evidence of a single DENV infection (25/52, 48.1%). In total, 17 of the 61 participants (27.9%) had NAbs against ZIKV and all four DENV serotypes, and an additional 9 (14.8%) had NAbs against ZIKV and DENV1, 2, and 3. NAbs against DENV2 were the most prevalent (44/61, 72.1%) followed by DENV3 (38/61, 62.3%) and DENV1 (36/61, 59.0%). Of note, anti-ZIKV NAbs were more prevalent than anti-DENV4 NAbs (27/61, 44.3% and 21/61, 34.4%, respectively). Primary ZIKV infection was detected in two participants, confirming that ZIKV co-circulates in this region. Thus, residents of DENV-endemic regions with repeated exposure to DENV have higher titers of NAbs against ZIKV than individuals with only a single DENV exposure.

## Introduction

Dengue virus (DENV) and Zika virus (ZIKV) are members of the *Flavivirus* genus, which includes other pathogens such as yellow fever virus, West Nile virus, and Japanese encephalitis virus (JEV). DENV and ZIKV are structurally similar, share some common transmission routes, such as the bite of infected *Aedes* species mosquitoes, and are prevalent in many overlapping regions of the world, including Thailand^1^. Indeed, a recent observational study suggested that ZIKV has circulated in Thailand at least since 2002^2^. In addition, a recent outbreak of ZIKV occurred in Asia^3,4^ but the prevalence was lower than the outbreaks in the Americas, particularly Latin America. In contrast, the incidence of DENV in Asia is estimated to be about double that in Latin America*.*

Although most DENV and ZIKV infections are asymptomatic, both viruses can have devastating and potentially life-threatening consequences. DENV, which exists as four serotypes (DENV1–4), can cause diverse symptoms ranging from mild fever to dengue hemorrhagic fever and fatal dengue shock syndrome, collectively known as severe dengue^5^. Similarly, ZIKV infections in adults may be associated with Guillain–Barré syndrome^6^, and infections in pregnant women can lead to severe congenital malformations, such as microcephaly, in the offspring^7^. These potential outcomes, together with the rapid regional spread of ZIKV, led the World Health Organization to declare ZIKV a public health emergency in February 2016^8^.

The convergence of several areas of work has amplified our need to understand the health ramifications of sequential infections with ZIKV or DENV1–4 heterotypes. First, the high sequence homology between ZIKV and DENV generates substantial antigenic overlap, leading to cross-reactive T and B cell immune responses*.*

Second, pre-existing immunity to one flavivirus or serotype has been shown to either protect against or exacerbate a subsequent heterologous virus infection, but the mechanisms controlling the particular outcome are unclear^9^*.* Three recent studies have reported that pre-existing anti-DENV immunity confers protection against subsequent ZIKV infection in humans^10–12^, and our studies with mouse models of flaviviral infections suggest that DENV-elicited CD8^+^ T cells may play a key role in mediating the cross-protection against ZIKV^13–15^. In vitro studies with cultured animal and human cells also suggest that ZIKV-cross-reactive DENV-elicited antibodies (Abs) can mediate Ab-dependent enhancement (ADE) of infection, providing a mechanism by which exposure to DENV might increase the severity of a subsequent ZIKV infection^16–21^. Conversely, there is strong evidence for ZIKV Ab-mediated DENV ADE from studies in mice, rhesus macaques, and humans. In mice, cross-reactive monoclonal Abs generated against ZIKV mediate DENV ADE^22^, inactivated ZIKV vaccination enhances DENV disease severity^23^, and we showed that maternally acquired ZIKV Abs cause severe dengue-like disease^24^. In rhesus macaques, prior ZIKV infection results in increased peak DENV viremia^25^. And in long-term human cohorts in Nicaragua, 1 prior infection of either ZIKV or DENV, followed by 1 ZIKV infection, elevated the risk of symptomatic DENV infection and disease severity^26^.

Third, ZIKV is continuing to spread in some DENV-endemic regions, where the majority of the population has already been exposed to DENV^27^, thereby increasing the potential for detrimental ADE of infection upon exposure to ZIKV or DENV heterotypes. Thus, it is essential that we understand whether and how pre-existing anti-DENV immunity may be deleterious or beneficial upon ZIKV infection in residents of DENV-endemic regions.

In this study, we examined serum samples from unselected healthy adults living in Nakhon Ratchasima, Thailand for the presence of anti-DENV1–4 and anti-ZIKV Abs, and we investigated the extent to which they could cross-neutralize heterologous virus infections in vitro. We found a striking association between repeated DENV infection and the presence of a strong and broad cross-neutralizing Ab (cross-NAb) response to ZIKV and DENV heterotypes.

## Results

### Seroprevalence of anti-DENV and anti-ZIKV IgG

Blood samples were collected from 64 participants and tested for anti-DENV IgG and anti-ZIKV E IgG by ELISA. Of the 64 donors, 61 were healthy Thai subjects who resided in Nakhon Ratchasima, Thailand, and were unselected for prior virus exposure or serostatus. Blood samples were collected from these individuals between April and July 2016, at which time ZIKV had been documented in the region for more than a decade^2^. The remaining three blood samples (negative controls) were obtained from European donors who had never traveled to Southeast Asia.

**Figure 1:**
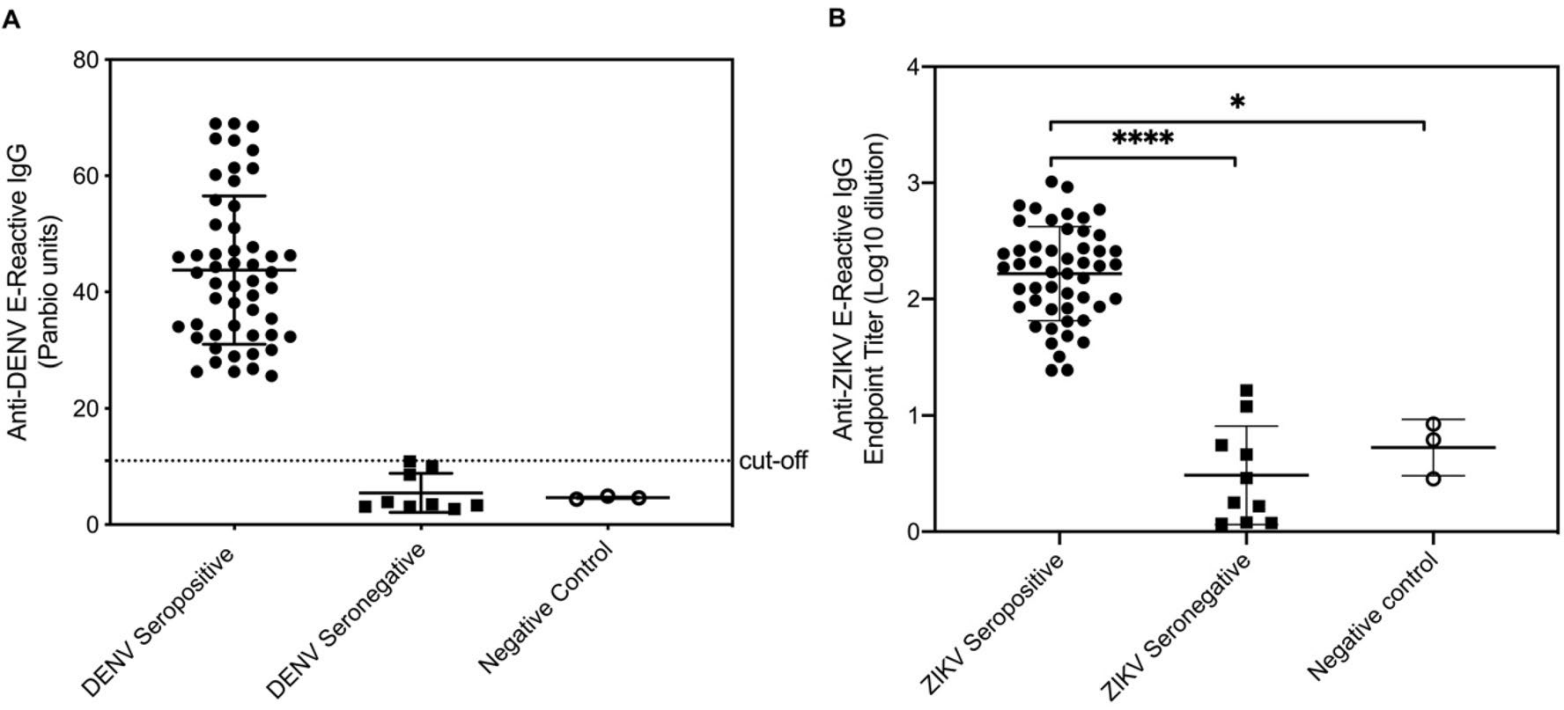
DENV and ZIKV seroprevalence in healthy adult residents of a DENV-endemic region in Thailand. **(A)** DENV E antigen-specific IgG was quantified by ELISA. Data are presented as the mean ± SD of 52 DENV seropositive and 9 DENV seronegative samples from Thai subjects and 3 samples from negative control subjects. **(B)** Endpoint titers of ZIKV E antigen-specific IgG were determined by ELISA. Data are presented as the mean ± SD of 51 ZIKV seropositive and 10 ZIKV seronegative samples from Thai subjects and 3 samples from negative control subjects. Each symbol represents an individual sample. *P < 0.05 and ****P < 0.0001 by the Kruskal–Wallis test.

Selected demographic features and the DENV and ZIKV serostatus of the 61 Thai subjects are shown in Table 1. The group consisted of 25 men and 36 women with a median age of 31 years (SD 9.9, range 18–69 years). Fifty-two subjects (85.3%) were seropositive for DENV IgG, indicating that most participants had been infected with DENV at least once ( Fig. 1A and Table 1). A breakdown of DENV seroprevalence indicated a positive association between prior exposure to DENV and age. Thus, 100% of participants aged 38 years and older were DENV seropositive (n = 21), compared with 80% of participants aged 28–37 years (n = 16) and 75% of those aged 18–27 years (n = 15) (Table 1). A total of 31 women (86.1%) and 21 men (84.0%) were DENV seropositive, indicating a lack of significant association between sex and seroprevalence. The three control sera were DENV seronegative, as expected (Fig. 1A).

**Table 1:** Characteristics and DENV/ZIKV serostatus of participants.

Parameter	All participants, *n* (%)	DENV IgG-positive, *n* (%)	ZIKV IgG-positive, *n* (%)
N (% of total)	61/61 (100.0)	52/61 (85.3)	51/61 (83.6)
**Sex**			
Male	25/61 (41.0)	21/25 (84.0)	20 (80.0)
Female	36/61 (59.0)	31/36 (86.1)	31 (86.1)
**Age (years)**			
18–27	20 (32.8)	15 (75.0)	14 (70.0)
28–37	20 (32.8)	16 (80.0)	16 (80.0)
38–47	17 (27.9)	17 (100.0)	17 (100.0)
≥ 48	4 (6.56)	4 (100.0)	4 (100.0)

Fifty-one of the 61 (83.6%) Thai subjects were ZIKV seropositive (Table 1), and as observed for DENV serostatus, there was no significant association between sex and ZIKV seroprevalence, with 86.5% of men (31/36) and 80.0% of women (20/25) being ZIKV seropositive. ZIKV seropositivity was positively associated with age: all 21 (100%) participants aged ≥ 38 years were ZIKV seropositive, compared with 16/20 (80%) aged 28–37 years and 14/20 (70%) of those aged 18–27 years (Fig. 1B and Table 1). Nine of the 61 individuals (4 men aged 19–31 years and 5 women aged 26–33 years) were seronegative for both DENV and ZIKV. Thus, of the 61 Thai subjects tested, 51 were DENV and ZIKV seropositive, 1 was seropositive for DENV only, and 9 were DENV and ZIKV naïve.

### Prevalence of anti-DENV1–4 and anti-ZIKV neutralizing antibodies

**Figure 2:**
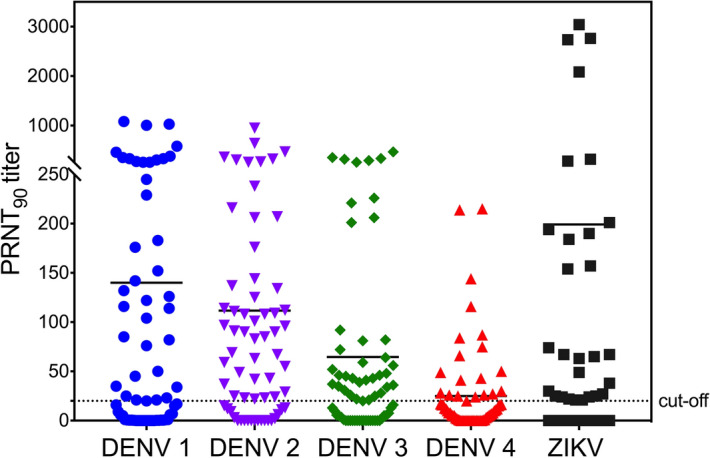
Distribution of neutralizing antibody titers against DENV 1–4 and ZIKV. Anti-DENV1–4 and ZIKV NAbs were measured by PRNT. The dashed line indicates PRNT_90_ = 20, which was the cutoff value for positive/negative stratification of sera. Each symbol represents an individual sample. Horizontal bar represents the mean. N = 61.

To determine whether the 61 serum samples from Thai subjects contained neutralizing activity, we screened the sera for anti-DENV1–4 and ZIKV NAbs using a plaque reduction neutralization test (PRNT) with LLC-MK_2_ cells. Neutralizing capacity is expressed as PRNT_90_ values (Fig. 2, Tables 2 and 3). Among the 61 sera, 25 (41.99%) contained NAbs against between one and three DENV serotypes, 17 (27.87%) contained NAbs against ZIKV and all four DENV serotypes, and 9 (14.75%) contained NAbs against ZIKV and between one and three DENV serotypes (Tables 2 and 3). Thus, nearly all of the sera contained NAbs against at least one DENV serotype and more than half of the sera contained anti-ZIKV NAbs. NAbs against DENV2 were the most prevalent (44/61, 72.1%) followed by DENV3 (38/61, 62.3%), DENV1 (36/61, 59.0%), and DENV4 (21/61, 34.4%).

**Table 2:** Characteristics of participants according to DENV/ZIKV-neutralizing antibody status.

	Prevalence of virus/serotype-specific neutralizing antibodies				
	DENV1, *n* (%)	DENV2, *n* (%)	DENV3, *n* (%)	DENV4, *n* (%)	ZIKV, *n* (%)
N (% of total)	36/61 (59.0)	44/61 (72.1)	38/61 (62.3)	21/61 (34.4)	27/61 (44.3)
**Sex**					
Male	15 (60.0)	18 (72.0)	15 (60.0)	7 (28.8)	10 (40.0)
Female	21 (58.3)	26 (72.2)	23 (63.9)	14 (38.9)	17 (47.2)
**Age (years)**					
18–27	10 (50)	13 (65.0)	10 (50.0)	7 (35.0)	8 (40.0)
28–37	13 (65.0)	13 (65.0)	11 (55.0)	4 (20.0)	7 (35.0)
38–47	10 (58.8)	14 (82.4)	13 (76.5)	8 (47.0)	9 (52.9)
≥ 48	3 (75.0)	4 (100.0)	3 (75.0)	2 (50.0)	3 (75.0)

Positive sera were defined as a 90% neutralizing titer > 20.

**Table 3:** Serostatus and neutralizing antibody profile of participants.

Serum number	PRNT_90_					Seropositive		Sex	Age (yr)	Hospitalized with dengue (yr)
	DV 1	DV2	DV3	DV4	ZV	DENV	ZIKV			
**Past primary DENV 1 (n = 7)**										
12	258	23	22	< 20	< 20	Pos	Pos	F	32	
13	356	67	28	< 20	< 20	Pos	Pos	M	32	
31	142	23	20	< 20	< 20	Pos	Pos	M	26	Twice:1997, 2007
34	332	59	< 20	< 20	38	Pos	Pos	M	27	
38	274	< 20	43	< 20	< 20	Pos	Pos	F	26	
40	1007	134	206	43	74	Pos	Pos	F	29	
51	152	22	< 20	< 20	< 20	Pos	Neg	M	29	
**Past primary DENV 2 (n = 13)**										
5	45	948	46	< 20	25	Pos	Pos	F	42	
7	< 20	238	46	27	< 20	Pos	Pos	F	47	
11	< 20	63	< 20	< 20	< 20	Pos	Pos	F	23	
14	< 20	111	< 20	< 20	< 20	Pos	Pos	F	31	
19	21	144	< 20	< 20	< 20	Pos	Pos	M	28	
21	82	642	31	84	22	Pos	Pos	F	24	Once: 2009
32	< 20	29	< 20	< 20	< 20	Pos	Pos	F	21	
33	< 20	49	< 20	< 20	< 20	Pos	Pos	F	22	
35	< 20	109	< 20	< 20	< 20	Pos	Pos	M	29	
46	< 20	24	< 20	< 20	< 20	Pos	Pos	F	56	
49	< 20	55	< 20	< 20	< 20	Pos	Pos	F	23	
58	< 20	42	< 20	< 20	< 20	Pos	Pos	F	44	
65	21	108	< 20	< 20	< 20	Pos	Pos	F	42	
**Past primary DENV 3 (n = 5)**										
6	23	85	294	28	< 20	Pos	Pos	F	42	
53	< 20	< 20	62	< 20	< 20	Pos	Pos	M	24	
59	< 20	< 20	35	< 20	< 20	Pos	Pos	F	45	
63	< 20	< 20	56	< 20	< 20	Pos	Pos	M	45	
64	< 20	< 20	43	< 20	< 20	Pos	Pos	M	45	Once, year N/A
**Past secondary DENV (n = 23)**										
1	122	91	21	< 20	< 20	Pos	Pos	M	41	
2	104	90	48	< 20	184	Pos	Pos	M	39	Once: 2000
3	245	206	201	214	276	Pos	Pos	F	41	Once, year N/A
4	229	324	470	215	30	Pos	Pos	F	44	
8	76	25	258	25	< 20	Pos	Pos	M	31	
9	126	112	59	< 20	49	Pos	Pos	M	30	
10	20	< 20	41	< 20	< 20	Pos	Pos	F	33	
20	< 20	305	< 20	116	65	Pos	Pos	M	46	Once, 1974
22	116	364	36	144	67	Pos	Pos	F	40	
23	462	125	37	26	154	Pos	Pos	F	28	
25	307	90	221	87	87	Pos	Pos	M	23	
26	331	216	226	24	24	Pos	Pos	F	26	
28	50	266	64	75	220	Pos	Pos	M	25	Once: 2003
37	259	137	320	49	27	Pos	Pos	F	49	
39	114	69	45	< 20	24	Pos	Pos	F	32	
42	370	83	25	< 20	190	Pos	Pos	M	31	
45	176	108	72	26	157	Pos	Pos	M	69	
47	35	96	26	66	25	Pos	Pos	M	33	
50	34	260	34	< 20	194	Pos	Pos	F	50	
52	1081	114	92	30	201	Pos	Pos	F	24	
57	< 20	97	81	< 20	320	Pos	Pos	F	43	
54	183	469	39	41	67	Pos	Pos	F	23	
61	132	< 20	335	< 20	< 20	Pos	Pos	F	32	
**Past primary ZIKV (n = 2)**										
15	< 20	< 20	< 20	< 20	281	Pos	Pos	F	37	
30	85	207	52	26	2735	Pos	Pos	M	26	
**Past secondary ZIKV (n = 2)**										
24	583	176	82	50	2084	Pos	Pos	F	38	Twice: 1983, 1986
60	1028	101	353	37	3040	Pos	Pos	F	39	
**Naïve (n = 9)**										
17	0	0	0	0	0	Neg	Neg	M	30	
18	0	0	0	0	0	Neg	Neg	M	24	
27	0	0	0	0	0	Neg	Neg	F	26	
29	0	0	0	0	0	Neg	Neg	M	31	
36	0	0	0	0	0	Neg	Neg	F	26	
41	0	0	0	0	0	Neg	Neg	M	19	
43	0	0	0	0	0	Neg	Neg	F	30	
44	< 10	0	0	0	0	Neg	Neg	F	26	
48	< 20	< 20	< 20	< 20	0	Neg	Neg	F	33	

*DV* DENV, *F* female, *m* male, *Neg* seronegative, *Pos* seropositive, *PRNT*_*90*_ 90% neutralizing titer in the PRNT assay, *yr* year, *ZV* ZIKV.

There were no significant differences in the proportion of sera from the 25 men and 36 women positive for NAbs; thus, ~ 59%, ~ 72%, ~ 62%, ~ 34%, and ~ 44% of sera from both sexes contained NAbs against DENV1, DENV2, DENV3, DENV4, and ZIKV, respectively. Not surprisingly, the prevalence of NAbs against each DENV serotype and ZIKV increased significantly with age. For example, the proportion of participants in each age group positive for anti-DENV1 NAbs increased from 50% (10/20) at age 18–27 years to 75% (3/4) by age ≥ 48 years, and a similar age-dependent increase was observed for NAbs against DENV2–4 and ZIKV (Table 2).

### Neutralizing antibody profiles according to DENV and ZIKV serostatus

**Figure 3:**
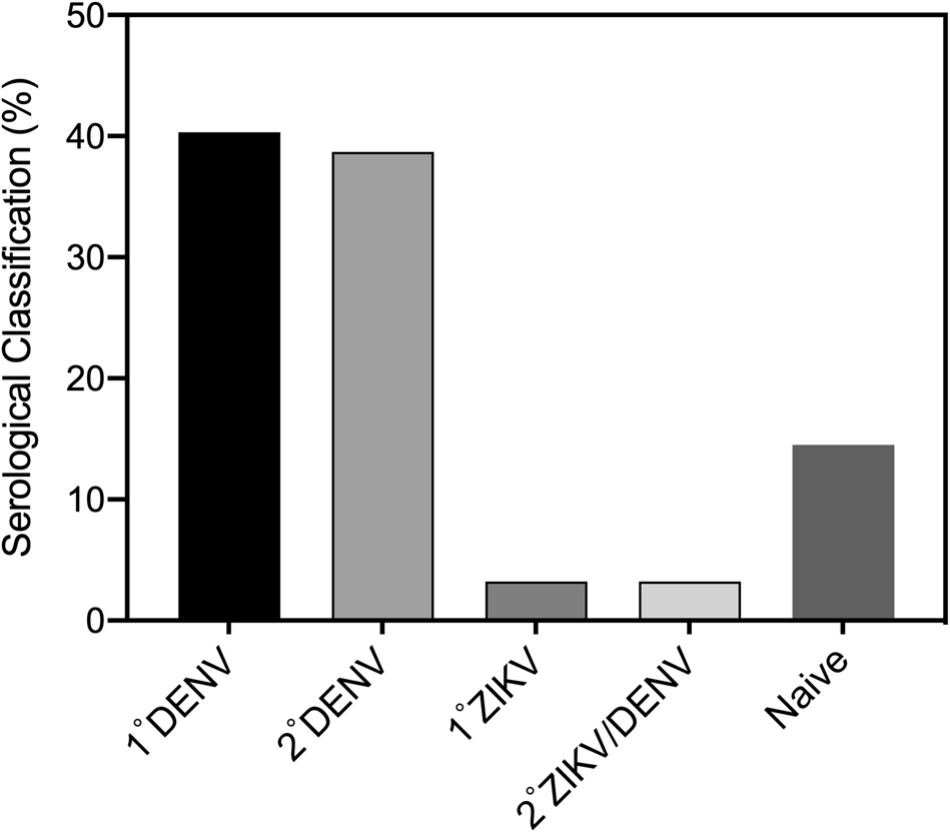
Distribution of sera representing primary and secondary DENV or ZIKV infection status. Seropositivity was defined NT_90_ ≥ 20. Primary (1°) and secondary (2°) infections with ZIKV and/or DENV were defined as described in the “Methods” section. The number of samples considered to represent 1° DENV infection, 2° DENV infection, 1° ZIKV infection, 2° ZIKV/DENV infection, and no prior infection (naïve) was 25, 23, 2, 2, and 9, respectively.

Next, we analyzed whether and how the prevalence of anti-DENV or anti-ZIKV NAbs was affected by prior exposure to DENV or ZIKV. Of the 61 samples, the serostatus of 25 (41.0%), 23 (37.7%), 2 (3.3%), 2 (3.3%), and 9 (14.8%) samples were indicative of a single (primary) DENV infection, multiple (secondary) DENV infection, primary ZIKV infection, secondary ZIKV/DENV infection, or no prior infection, respectively (Fig. 3). Among the 25 participants whose serology was consistent with primary DENV infection, most were DENV2 infections, followed by DENV1, and DENV3 (n = 13, 7, and 5, respectively). In contrast, none of the serological profiles were consistent with a primary DENV4 infection (Table 3). Only 4 of the 25 (16%) participants (no. 40, 34, 21, and 5) with primary DENV infection had high NAb titers against DENV, but they exhibited cross-neutralization of ZIKV (Table 3), suggesting a correlation between the presence of anti-ZIKV NAbs and a high level of anti-DENV NAbs. In contrast, 19 (82.6%) of the 23 participants with secondary DENV infection had anti-ZIKV NAbs. The four negative samples had low titers of NAbs against DENV1–4, suggesting that the donors mounted weak NAb responses to all flaviviruses/serotypes tested. Thus, nearly all of the participants showing evidence of repeated infection with DENV contain Abs with robust ZIKV-neutralizing capacity*.* Only two participants showed serological evidence of primary ZIKV infection. One of these (no. 15) had a low PRNT_90_ for ZIKV and was negative for DENV NAbs, and the second (no. 30) had a very high titer of ZIKV NAbs and lower but marked neutralizing activity against all four DENV serotypes. These results indicate that repeat exposure to heterotypic DENV elicits a strong cross-NAb response against ZIKV.

### ZIKV-neutralizing activity of DENV-elicited antibodies

**Figure 4:**
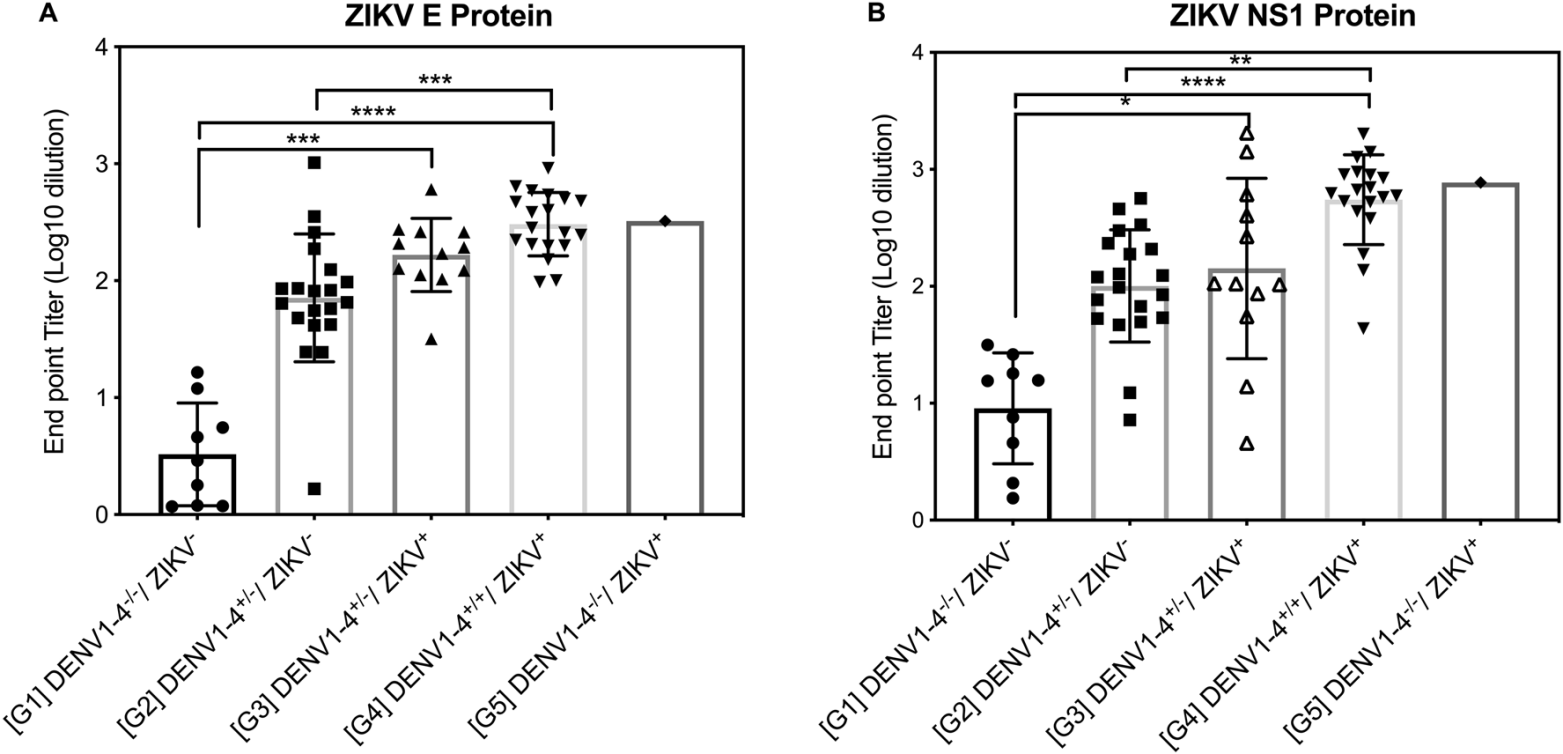
Endpoint titers in anti-ZIKV E and NS1 protein ELISAs for sera grouped by neutralizing activity. IgG against ZIKV E protein **(A)** and ZIKV NS1 protein **(B)** were quantified by ELISA for the 61 sera grouped according to the presence or absence of neutralizing antibodies against DENV1–4 and ZIKV as follows. Group 1 (n = 9), negative for all DENV serotypes and ZIKV; G2 (n = 20), positive for between one and three DENV serotypes and negative for ZIKV; G3 (n = 12), positive for between one and three DENV serotypes and positive for ZIKV; G4 (n = 19), positive for all DENV serotypes and positive for ZIKV; G5 (n = 1), negative for all DENV serotypes and positive for ZIKV. Data are presented as the mean ± SD, with each symbol representing an individual sample. *P < 0.05, **P < 0.01, ***P < 0.001, ****P < 0.0001 by the Kruskal–Wallis test.

The 61 sera were assigned to one of six groups according to their DENV1–4 and/or ZIKV NAb serostatus and analyzed for their reactivity to ZIKV NS1 and E proteins: group 1, DENV^−^ ZIKV^−^ (n = 9); group 2, DENV^+^ (one to three serotypes) ZIKV^–^ (n = 20); group 3, DENV^+^ (one to three serotypes) ZIKV^+^ (n = 12); group 4, DENV^+^ (all four serotypes) ZIKV^+^ (n = 19); and group 5, DENV^−^ ZIKV^+^ (n = 1). Notably, all of the DENV seropositive samples also contained anti-ZIKV E (Fig. 4A) and/or anti-ZIKV NS1 (Fig. 4B) Abs, and the binding patterns for ZIKV E and NS1 were similar for each group. Although group 2 Abs were defined as negative for anti-ZIKV NAbs, Abs against ZIKV E and NS1 antigens could be detected. This result might be due to cross-reactivity by anti-DENV Abs. The anti-ZIKV Ab endpoint titer of sera with NAbs against all four DENV serotypes (group 4) was not significantly higher than that of the sera with NAbs against fewer DENV serotypes (group 3) (*P* > 0.99), indicating that the anti-DENV NAb response influenced the magnitude of the cross-reactive anti-ZIKV Ab response.

**Figure 5:**
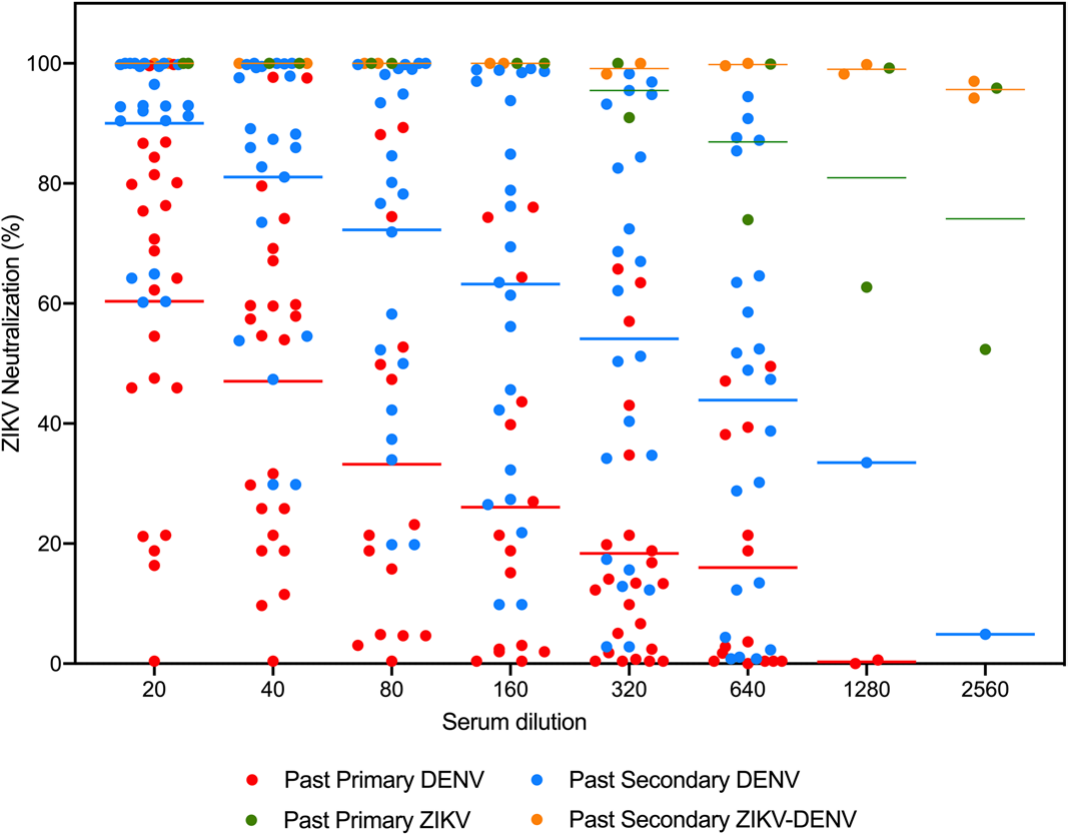
ZIKV-neutralizing activity of serum samples. Comparison of ZIKV-neutralizing activity in subjects with serostatus indicative of past primary DENV serotype 1, 2, or 3 (red dot), past secondary DENV (blue dot), past primary ZIKV (green dot), and past secondary ZIKV and DENV (orange dot) infection*.* Each dot represents an individual sample. Horizontal bar represents the mean (N = 61).

We then compared ZIKV cross-neutralizing activity between sera from the past primary and past secondary DENV infection groups, and we found that higher titers of ZIKV cross-NAbs correlated with past secondary DENV infection serostatus (Fig. 5). Collectively, these results indicate that both the quantity and quality of DENV and ZIKV NAbs are influenced by prior exposure to the heterologous virus/serotype.

## Discussion

In this study, we sought to determine whether healthy residents of a DENV-endemic region in Thailand showed evidence of cross-NAbs to ZIKV. DENV has been endemic in the area since 1958^28^, and although there have been no outbreaks of ZIKV in Thailand, its presence has been documented since 2002^2^. These observations suggest that most residents of the region have likely been infected with DENV, if not ZIKV, at least once. This suggestion is supported by our results here showing that 52 of the 61 participants in our study were DENV seropositive, 25 of whom showed evidence of repeat infections based on NAbs. Similarly, 51 participants were ZIKV seropositive, 4 of whom were confirmed as having past primary or past secondary ZIKV infection based on NAbs. Thus, a substantial proportion of the participants showed evidence of exposure to DENV but not ZIKV, yet their sera contained strongly cross-neutralizing activity against ZIKV.

In the present study, 44.3% (27/61) of serum samples contained anti-ZIKV NAbs (based on PRNT90 ≥ 20), which was a considerably higher frequency (22.2% based on PRNT90 ≥ 20) than that detected in a study of 135 adults in Bangkok, Central Thailand^29^. The authors of that study suggested that protection against ZIKV arises from prior exposure to ZIKV, not DENV, based on the finding that Abs to ZIKV NS1 were detected only in serum samples with PRNT_90_ ≥ 20 and not those with PRNT_90_ < 20 for ZIKV NAbs^30^. This discrepancy could be due to the different regions in Thailand from which the samples were obtained, together with the higher circulation of DENV compared with ZIKV throughout the country. Although the population density of Nakhon Ratchasima is much lower than that of Bangkok (129 vs 3592 people/km^2^), public health access and preventive measures such as mosquito nets are less accessible to residents of the more sparsely populated areas. Thus, the relative exposure to DENV is likely higher and exposure to ZIKV is likely lower for residents of Nakhon Ratchasima compared with Bangkok^2^. In support of this hypothesis, a 2019 study of field-caught mosquitoes by Phumee and colleagues found a lower prevalence of ZIKV-carrying mosquitoes in the Northeastern region of Thailand (which includes Nakhon Ratchasima) than in the Central region (including Bangkok)^31^. The results of the present study thus indicate that ZIKV seroprevalence levels in Thailand are most likely dependent on geography, and that multiple exposures to DENV of residents of the Northeastern region likely results in acquisition of ZIKV cross-NAbs.

Collins et al. studied individuals from North Carolina who traveled to DENV- or ZIKV-endemic countries and returned with confirmed DENV or ZIKV infections^32^. They found that patients with primary DENV infection showed no evidence of ZIKV NAbs, and only a low frequency of those with secondary DENV infection had ZIKV NAbs (23%, based on the IC_50_ in a neutralization assay). That study was performed with samples collected ≥ 6 months after infection, suggesting that the NAb response had likely become more specific to a particular virus/serotype^32^. The discrepancy in findings between our study and that of Collins et al. may be due to differences in residency (Thailand *vs* United States) and/or the time elapsed after primary infection (undefined *vs* ≥ 6 months). Recent cohort studies of patients from Latin America and Asia, including Thailand, showed that convalescent sera collected ≥ 5 months after secondary DENV infection failed to cross-neutralize ZIKV^33^*.* In contrast, our results support the findings of a previous study that healthy but repeatedly infected residents of regions hyperendemic for DENV can maintain a repertoire of broadly cross-NAbs against the four DENV serotypes^34^. Furthermore, our results are consistent with a previous cohort study of patients with confirmed dengue in Southern Vietnam, which showed that individuals with multiple exposures to DENV carried higher cross-NAb titers to ZIKV than individuals who had been exposed to DENV once^35^. A recent study of memory B cells from dengue patients in Nicaragua showed that DENV infection induced ZIKV cross-reactive memory B cells, particularly in patients with multiple exposures to DENV. Moreover, the ZIKV cross-neutralizing capacity of Abs generated by the memory response was higher than that of NAbs generated after primary DENV infection^36^. Another study suggested that cross-reactive immunity resulting from prior ZIKV exposure was responsible for the decline in dengue incidence in the Americas in 2017^37^. These results suggest that both the number and sequence of prior ZIKV and/or DENV serotype infections influence the risk of severe dengue disease^26^. Interestingly, recent study of ZIKV phylogenetic and in silico analysis indicated that while DENV and ZIKV share immunodominant B cell epitopes, ZIKA T-cell epitopes were derived from *Culex*-borne flaviviruses such as JEV, which might induce cross-protective T-cell responses against ZIKV. This could explain why ZIKA did not cause major epidemics in Thailand and other Asian countries where JEV is widespread^38^.

In summary, our study provides insights into the prevalence and potency of anti-DENV1–4 and ZIKV cross-NAbs in healthy adults living in a DENV-endemic region of Thailand. We found that an equally high proportion of participants (~ 85%) were DENV or ZIKV seropositive. Nearly all of the DENV seropositive individuals showed evidence of cross-NAbs against ZIKV and/or heterologous DENV serotypes, and about two-thirds of ZIKV-seropositive individuals had NAbs against at least one DENV serotype. All participants with secondary DENV infection contained anti-ZIKV E- and NS1-reactive Abs. Collectively, these results show that repeated exposure to DENV1–4 elicits an Ab response that strongly cross-neutralizes heterologous DENV serotypes as well as ZIKV. These observations not only have important implications for the surveillance of residents of DENV-endemic regions where ZIKV co-circulates, and vice versa, but also inform the design of safe, effective, and potentially cross-protective vaccines.

## Methods

### Study participants and sample collection

The study included blood samples collected from 61 healthy volunteers (25 men, 36 women, aged 18–69 years) who were seen at the Suranaree University of Technology (SUT) Hospital, Nakhon Ratchasima, Thailand. The inclusion criteria were no prior hospitalization or hospitalization > 5 years ago for DENV infection. All subjects were residents of Nakhon Ratchasima, were enrolled nonconsecutively between April and July of 2016, and provided a single (5 mL) blood sample. The cohort demographics are representative of the population in the Northeastern region of Thailand. As controls, blood samples were also obtained from three female healthy European volunteers (mean age 22 years) who had never traveled to Southeast Asia and were confirmed here to be ZIKV and DENV seronegative. The study was performed in accordance with the Declaration of Helsinki. The study protocol was approved by the Ethics Committee of SUT (protocol no. EC-59–10) and the Institutional Review Board of the La Jolla Institute for Immunology (protocol no. IB-189–1118). Written informed consent was obtained from all participants before blood collection.

### Virus propagation

World Health Organization reference strains DENV1 16007, DENV2 16681, DENV3 16562, and DENV4 1036 were kindly provided by Dr. Duncan R. Smith (Mahidol University, Thailand). ZIKV Asian lineage strain SD001 was isolated from a donor who had visited Venezuela in 2016^39^. All viral stocks were propagated in *Aedes albopictus* C6/36 cells (ATCC no. CRL-1660). Virus-containing supernatants were clarified by low-speed centrifugation, concentrated by ultracentrifugation, and supplemented with fetal bovine serum to a final concentration of 20%. All viral stocks were stored at − 80 °C. Viral titers were determined using a focus forming assay with baby hamster kidney (BHK)-21 cells (ATCC no. CCL-10) as previously described^40^.

### In vitro neutralization assay

To quantify anti-DENV1–4 and anti-ZIKV neutralizing Abs (NAbs), a plaque reduction neutralization test (PRNT) was performed with LLC-MK_2_ cells as previously described^29^. For ZIKV NAbs, the standard PRNT was modified by using DMEM (Capricorn Scientific, Germany) containing 1% FBS and 0.75% NaHCO_3_ as the sample and virus diluent solution. Inoculated cells were gently overlaid with 2X nutrient and 1.6% UltraPure agarose (Invitrogen, Thermo Fisher Scientific, Carlsbad, CA) and maintained at 37 °C in a 5% CO_2_ atmosphere for 7 days. Plaque numbers after incubation with (test) or without (control) test sera were counted and the percentage neutralization was calculated as 100 × ([control plaque number – test plaque number]/control plaque number). The World Health Organization recommendation for DENV PRNT suggests that an PRNT_90_ cutoff value should be used for samples obtained in flavivirus-endemic areas^41^. For both viruses, PRNT_90_ was defined as the reciprocal of the serum dilution resulting in a 90% reduction of viral infectivity. PRNT_90_ was calculated by non-linear regression using Prism 8 software (GraphPad, La Jolla, CA).

### DENV and ZIKV ELISA

Serum levels of IgG against DENV E antigen serotypes (mixture of 1, 2, 3, and 4) were quantified using an indirect ELISA kit (E-DEN-01G, Panbio, Brisbane, QLD, Australia), according to the manufacturer’s instructions. DENV-specific IgG is expressed as Panbio units, which were calculated as: ([optical density; OD value of the test specimen]/[mean cutoff calibrator OD value] × 10). According to the kit manufacturers’ recommendations, samples with > 11 Panbio units were considered positive. Serum levels of IgG against recombinant ZIKV E and NS1 proteins were quantified by ELISA, as previously described^40^. Anti-ZIKV IgG levels are expressed as the endpoint titer, calculated as the reciprocal of the highest serum dilution giving an OD value ≥ 2-fold the OD value of the blank sample (bovine serum albumin in phosphate-buffered saline)*.* Anti-ZIKV E IgG positivity was defined as endpoint titers > 1 standard deviation (SD) above the value for the serum negative control value.

### Serostatus definitions

To ensure robust results, we set a definition of positive results in the NAb assays to NT_90_ ≥ 20 (i.e., 90% neutralization by ≥ 20-fold serum dilution). NAb titers and reactivities were used to identify sera as representing single (primary infection) or multiple (secondary infection) prior infections, as previously described^32,33^. Primary infection was defined as (i) an PRNT_90_ ≥ 20 against a single DENV serotype and an PRNT_90_ < 20 titer against all other DENV serotypes, (ii) an PRNT_90_ ≥ 20 against ZIKV and an PRNT_90_ < 20 titer against all DENV serotypes, or (iii) a dominant response against a single DENV serotype or against ZIKV, with an PRNT_90_ ≥ fourfold higher than the PRNT_90_ against the remaining virus/serotypes. Secondary infection was defined as (i) an PRNT_90_ ≥ 20 against both viruses or ≥ 2 DENV serotypes, and (ii) the dominant response had an PRNT_90_ ≤ fourfold higher than the PRNT_90_ of the next highest response.

### Statistical analysis

Data are expressed as the mean ± SD. Differences between group means were analyzed by two-way analysis of variance or the Kruskal–Wallis test. *P* < 0.05 was considered significant.
